# Seasonal variation of soil respiration in a tropical coastal shelter forest and its responses to environmental factors

**DOI:** 10.3389/fpls.2025.1747744

**Published:** 2026-01-14

**Authors:** Haihui Chen, Leyu Tian, Yiqing Chen, Zongzhu Chen, Shouqian Nong, Zhipan Lin, Xiangling Lei, Junting Jia, Shaofeng Su

**Affiliations:** 1 Hainan Academy of Forestry, Hainan Academy of Mangrove, Haikou, China; 2 Hainan Wenchang Forest Ecosystem Observation and Research Station, Wenchang, China; 3 Key Laboratory of Tropical Forestry Resources Monitoring and Application of Hainan Province, Haikou, China; 4 The Innovation Platform for Academicians of Hainan Province, Haikou, China

**Keywords:** annual carbon flux, carbon cycle, forest stand, seasonal variation, soil carbon pool, temperature sensitivity

## Abstract

**Introduction:**

Tropical coastal shelter forests exhibit unique environmental characteristics and material cycling processes. However, the seasonal dynamics and environmental drivers of soil respiration in these ecosystems remain poorly understood. This study investigated the spatial variation in soil respiration rates across typical tropical coastal shelter forest stands, characterized their seasonal patterns, clarified the roles of soil temperature and soil moisture, and identified the responses of soil respiration to environmental factors during the dry and wet seasons.

**Methods:**

Four typical tropical coastal shelter forests in Wenchang, Hainan, *Casuarina equisetifolia* forest (CA), *Cocos nucifera* forest (CO), mixed forest (MF), and secondary forest (SF), were monitored through monthly soil respiration measurements over one year. By integrating soil temperature, soil moisture, soil chemical properties, and climatic variables, we analyzed spatiotemporal variations in soil respiration rates and their environmental controls using regression analysis and a random forest model.

**Results:**

Soil respiration rates showed pronounced spatial variation among forest types, with the SF stand exhibiting substantially higher soil respiration and annual CO_2_ emissions than the others. Soil respiration, soil temperature, and soil moisture all displayed clear seasonal patterns, with markedly higher values during the wet season (May–October) than during the dry season (November–April). Soil temperature was the dominant driver of seasonal variation, showing a highly significant exponential positive relationship with soil respiration and explaining 57.2–84.8% of its variation. In contrast, soil moisture had much weaker explanatory power (1.8–14.4%), although temperature and moisture exhibited synergistic effects. Soil organic matter was identified as a key factor underlying spatial variation across stands, whereas seasonal changes in precipitation and soil pH contributed significantly to differences between the dry and wet seasons.

**Discussion:**

These findings improve our understanding of carbon cycling processes in tropical coastal shelter forests and provide a scientific basis for adaptive forest management under global climate change. The pronounced spatial variation in soil respiration among forest types highlights the importance of species composition and stand structure in regulating coastal carbon budgets. Future management strategies should incorporate these elements to enhance carbon sequestration and strengthen ecosystem resilience to climatic stressors.

## 1 Introduction

In the context of global climate change, the greenhouse effect, driven by a continuous increase in atmospheric CO_2_ concentrations, has emerged as a central challenge to global ecological security (IPCC, 2021). Soil respiration is a key component of the terrestrial carbon cycle and represents the dominant source of ecosystem respiration. It also plays a decisive role in regulating the terrestrial carbon balance through dynamic exchanges with atmospheric carbon pools (Schlesinger and Andrews, 2000; Hashimoto et al., 2015, 2023; Friedlingstein et al., 2022). Because of its large contribution to the global carbon budget, even minor fluctuations in soil respiration can substantially affect atmospheric CO_2_ concentrations (IPCC, 2021), making it an essential regulatory factor in climate change studies.

Soil respiration comprises two main components: heterotrophic respiration, produced by microbial decomposition of soil organic matter, and autotrophic respiration, derived from root metabolic activity (Bond-Lamberty et al., 2004; Hanson et al., 2000). Their temporal dynamics are regulated by a combination of abiotic and biotic factors. Abiotic factors, including climate conditions, soil temperature, soil moisture, and soil physicochemical properties, influence respiration fluxes by modulating microbial and root physiological processes. In contrast, biotic factors, such as vegetation type, microbial activity, and community composition, shape respiration through their effects on litter input, root activity, and microbial community structure (Gao et al., 2018; Li et al., 2021; Bond-Lamberty et al., 2024; Moyano et al., 2013; Qu et al., 2023; Hursh et al., 2017).

Forest ecosystems are major components of the terrestrial biosphere, and more than 40% of terrestrial organic carbon is stored in forest soils (Mayer et al., 2020). Therefore, soil respiration in forests plays an essential role in regulating the global carbon balance (Bond-Lamberty and Thomson, 2010). Numerous studies have examined soil respiration in forests in relation to climate change (Deng et al., 2018; Liang et al., 2024), human disturbance (Cheng et al., 2023; Yang et al., 2024), and differences among forest types (Gao et al., 2018; Guan et al., 2024). The effects of environmental factors on soil respiration in forests have been extensively explored (Gao et al., 2018; Li et al., 2021; Cusack et al., 2023; He et al., 2024). However, these studies focus primarily on non-coastal tropical forests or inland forest ecosystems. Systematic investigations into soil respiration dynamics in tropical coastal shelter forests, which are characterized by extreme environmental pressures (such as salinization and typhoon disturbances) and pronounced dry–wet seasonal transitions, remain scarce (Chen et al., 2017; Nong et al., 2025). As vital carbon sink functional zones in coastal regions, tropical coastal shelter forests have unique carbon cycling processes shaped by harsh and fluctuating habitats that cannot be fully inferred from inland forest studies. Moreover, different forest types within these ecosystems, from monoculture plantations to mixed and secondary forests, are likely to exhibit distinct soil respiration patterns owing to differences in species composition, canopy structure, root biomass, and litter quality. Therefore, comparing these forest types is essential for identifying stand characteristics and management practices that optimize carbon sequestration while maintaining ecosystem resilience to environmental stress. Targeted research on soil respiration dynamics in these forests is crucial to fill the knowledge gap in regional carbon budget assessments under global climate change.

Since the 1950s, the Chinese government has implemented large-scale coastal shelter forest programs to protect existing secondary forests in coastal zones while establishing monoculture plantations of species such as *Casuarina equisetifolia* and *Cocos nucifera* (Chen et al., 2017). Coastal shelter forests act as ecological barriers in tropical coastal regions and provide multiple ecological functions, including windbreak and sand fixation (Tucker et al., 2025), coastal protection (Chang and Mori, 2021; Zhu et al., 2024), and biodiversity maintenance (Vandermeer et al., 2000; Fortunel, 2023). Soil systems supporting these functions operate in unique and stressful environments characterized by long-term pressures, such as high temperature, high humidity, salinization, and typhoon disturbances, as well as pronounced seasonal variations. These conditions shape distinct seasonal characteristics of carbon cycling processes (Fan et al., 2021; Jia et al., 2023). Existing studies on these forest types in China have primarily examined windbreak and disaster resistance (Paz et al., 2018; Ibanez et al., 2024), carbon sink assessments (Fan et al., 2021), and stoichiometric characteristics (Nong et al., 2025). However, a critical gap remains, that is, few studies have systematically linked seasonal variations in soil respiration to their key environmental drivers. This gap limits the accuracy of carbon cycling models for China’s tropical coastal zones under climate change.

We conducted monthly soil respiration measurements over one year at the Wenchang Forest Ecosystem Observation and Research Station. Four typical tropical coastal shelterbelt types were selected as research objects: *Casuarina equisetifolia* forest (CA), *Cocos nucifera* forest (CO), mixed forest (MF), and secondary forest (SF). This study aimed to address the following questions. (1) What are the characteristics and differences in soil respiration among these tropical coastal shelterbelts? (2) How does soil respiration vary across different seasons? (3) What are the key environmental factors driving seasonal and spatial variations in soil respiration rates? Addressing these questions will improve our understanding of carbon cycling processes in tropical coastal shelterbelts and provide a scientific basis for forest management under changing climatic conditions.

## 2 Materials and methods

### 2.1 Overview of the research area

The research area is located in Wenchang City, Hainan Province (19°21’N–20°01’N, 110°28’E–111°03’E) and is characterized by a tropical maritime monsoon climate. The annual average air temperature is 23.9°C, with the coldest months occurring from December to February. The region receives 1600–1800 mm of rainfall annually and shows distinct wet and dry seasonal patterns. The wet season extends from May to October and contributes approximately 80% of the annual precipitation, whereas the dry season lasts from November to April of the following year (Nong et al., 2025). The study area is located on a coastal plain terrace with generally low elevations, averaging only 42.55 m above sea level. The dominant soil type is sandy loam formed from coastal sediments, which is characterized by acidic conditions and low nutrient availability (Chen et al., 2017; Nong et al., 2025).

### 2.2 Plot setup

**Figure 1:**
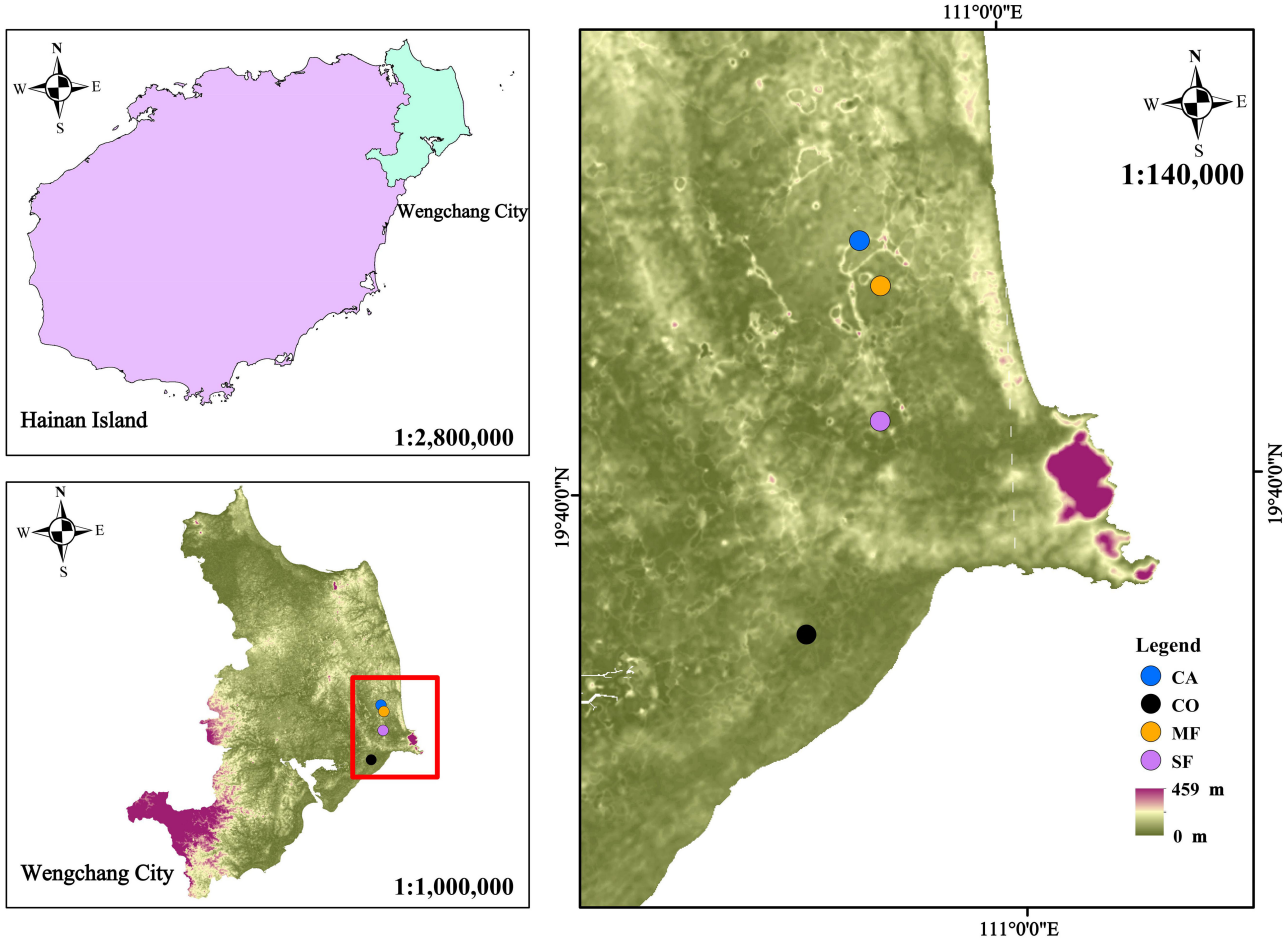
Location of the research area and distribution of sampling plots. CA, CO, MF, and SF represent *Casuarina equisetifolia* forest, *Cocos nucifera* forest, mixed forest, and secondary forest, respectively. The same abbreviations are used in **Figures 2**-**7**.

Within the four 1-hectare fixed monitoring plots representing CA, CO, MF, and SF at the Wenchang Forest Ecosystem Observation and Research Station, three 20 m × 20 m quadrats were randomly established in each plot with a minimum spacing of 20 m between quadrats. The locations of the quadrats are shown in **Figure 1**, and the stand characteristics of each forest type are summarized in **Table 1**.

**Table 1:** Site characteristics of the four tropical coastal shelter forest stands.

Indicator	Stand			
	CA	CO	MF	SF
Longitude	110°57’29” E	110°56’20” E	110°57’50” E	110°57’44” E
Latitude	19°44’00” N	19°37’35” N	19°43’15” N	19°41’02” N
Soil type	Sandy loam	Sandy loam	Sandy loam	Sandy loam
Tree height (m)	10.06 ± 0.25	9.87 ± 0.33	11.57 ± 2.78	9.26 ± 3.72
Diameter at breast height (cm)	18.01 ± 0.36	24.24 ± 1.54	19.64 ± 3.54	18.03 ± 5.34
Primary tree species	*Casuarina equisetifolia*	*Cocos nucifera*	*Pinus caribaea;* *Casuarina equisetifolia;* *Eucalyptus tereticornis;* *Acacia auriculiformis*	*Castanopsis wenchangensis;* *Calophyllum inophyllum;* *Litsea pseudoelongata;* *Symplocos poilanei;* *Heptapleurum heptaphyllum*

CA, CO, MF, and SF represent *Casuarina equisetifolia* forest, *Cocos nucifera* forest, mixed forest, and secondary forest, respectively. The same abbreviations are used in **Tables 2**-**5**.

### 2.3 Soil respiration rate and related factor determination

In each plot, six PVC soil respiration collars (20 cm in diameter and 10 cm in height) were installed in an S-shaped pattern, resulting in a total of 18 collars per forest plot. Each collar was inserted 5 cm into the soil to ensure stability during the experiment. Four small perforations (0.5 cm in diameter) were drilled into the buried portion of each collar to allow normal exchange of water and heat between the inside and outside soils. The first measurement of soil respiration rate was conducted approximately one week after collar installation to minimize disturbance effects. Before each measurement, all living plants inside the collars were removed. From early September 2023 to early August 2024, soil respiration rate was measured using an open-circuit automated soil carbon flux system (LI-8100A; LI-COR Inc., Lincoln, NE, USA). The period from 9:00 to 11:00 a.m. is considered optimal for estimating daily CO_2_ emissions (Xu and Qi, 2001). Therefore, measurements were conducted between 9:00 and 11:00 a.m. on two to three sunny days during the first half of each month, with each collar being recorded for 2 min. Soil temperature at a depth of 10 cm near each collar was measured using a digital handheld thermometer (AM-11T, Avalon, USA), and soil moisture at a depth of 0–10 cm was determined using a time-domain reflectometer (TDR300, Spectrum, USA).

### 2.4 Environmental factor determination

#### 2.4.1 Soil factor determination

In December 2023 (dry season) and July 2024 (wet season), soil samples were collected from each plot using a five-point sampling method (four corners and the center). Samples were collected from the 0–10 cm topsoil layer using a 5-cm-diameter soil auger. After plant roots and stones were removed, the soil was thoroughly homogenized, sealed in labeled bags, and transported to the laboratory. The samples were air-dried in the shade and passed through a 100-mesh sieve before analysis. Soil pH was measured potentiometrically at a soil-to-water ratio of 1:2.5. Soil organic matter (SOM) and soil organic carbon (SOC) were quantified using the potassium dichromate oxidation method. Total nitrogen (TN) was determined using the semi-micro Kjeldahl method, and total phosphorus (TP) was measured using acid-soluble molybdenum–antimony colorimetry. Available nitrogen (AN) was quantified using potassium chloride extraction followed by flow analysis, and available phosphorus (AP) was determined using sodium bicarbonate extraction combined with molybdenum–antimony anti-absorption photometry (Tan et al., 2020).

#### 2.4.2 Climatic factor determination

An automatic weather station (FIM.MEM09, Beijing Global Forest Company, China) was installed at each stand to record real-time precipitation (PRCP), air temperature (TEMP), and relative humidity (RH).

### 2.5 Data processing and analysis

The data were initially processed and statistically summarized using Microsoft Excel 2024, and subsequent analyses were performed using Origin 2024. Linear regression was used to model the relationship between soil respiration and soil moisture (Equation 1). Univariate exponential regression was applied to characterize the relationship between soil respiration and soil temperature (Equation 2). In addition, bivariate exponential regression was used to evaluate the combined effects of soil temperature and soil moisture on soil respiration (Equation 3) (Peng et al., 2009; Zhou et al., 2007). Pearson correlation analysis between soil respiration rates and environmental variables was conducted using the Correlation Plot App. Comparisons were performed for the dry (November–April) and wet (May–October) seasons based on the seasonal mean values of soil respiration for each period.

(1) Rs  =m+nW

(2) $$\text{Rs }=\text{a}e^{bT}$$

(3) $$\text{Rs }=\text{a}e^{bT}W^{\text{c}}$$

where *R_s_* denotes the soil respiration rate (μmol CO_2_·m^−2^ s^−1^); *T* and *W* represent the soil temperature (°C) at 10 cm depth and the soil moisture (%) in the 0–10 cm layer, respectively; and *m*, *n*, *a*, *b*, and *c* are undetermined coefficients.

The temperature sensitivity of soil respiration rate was expressed using *Q*_10_ (Equation 4):

(4) $$\text{Q}10\;=e^{10b}$$

where *b* is a parameter in Equation 2.

The annual soil respiration flux is expressed as *R* (gC·m^2^ a^−1^) (Equation 5):

(5) $$\text{R}=\sum_{\text{d}}ae^{bT}3600\times 24\times 12\times 10^{-6}$$

where *T* is the daily soil temperature at a depth of 10 cm, and *b* is the parameter in Equation 2. Regression equations (**Table 2**) were established between the monthly average soil temperature *T*_1_ at each plot and the monthly average air temperature *T*_2_ recorded by the meteorological station. Daily air temperature data were substituted into these equations to estimate the daily soil temperature *T* for each plot, which was then used to compute the annual flux.

**Table 2:** Regression equations between monthly average soil temperature and monthly average air temperature.

Stand	Regression equation	*R*2	*P*
CA	*T*2 = 1.067 *T*1 - 1.078	0.807	<0.001
CO	*T*2 = 1.475 *T*1 - 10.220	0.731	<0.001
MF	*T*2 = 1.329 *T* 1- 6.566	0.826	<0.001
SF	*T*2 = 1.043 *T*1 - 1.940	0.729	<0.001

*T*_1_ represents the monthly average soil temperature observed in the plot, and *T*_2_ denotes the monthly average air temperature recorded at the meteorological station.

SPSS 27 was used to perform repeated-measures ANOVA to assess the interactive effects of forest type and month on soil respiration, soil temperature, and soil moisture. To further examine differences among forest types in soil respiration, temperature, moisture, annual CO_2_ flux, *Q*_10_ values, and other environmental variables, one-way ANOVA (α = 0.05) followed by the LSD *post hoc* test was conducted.

Using the seasonal mean soil respiration values for the dry (November–April) and wet (May–October) seasons, we applied a random forest model to evaluate the relative importance of soil physicochemical and climatic variables, including pH, SOM, SOC, TN, TP, AN, AP, PRCP, TEMP, and RH. Random forest analysis and variable importance estimation were conducted using the randomForest package in R version 4.5.1.

## 3 Results and analysis

### 3.1 Soil respiration rate, soil temperature, and soil moisture across different stands

**Figure 2:**
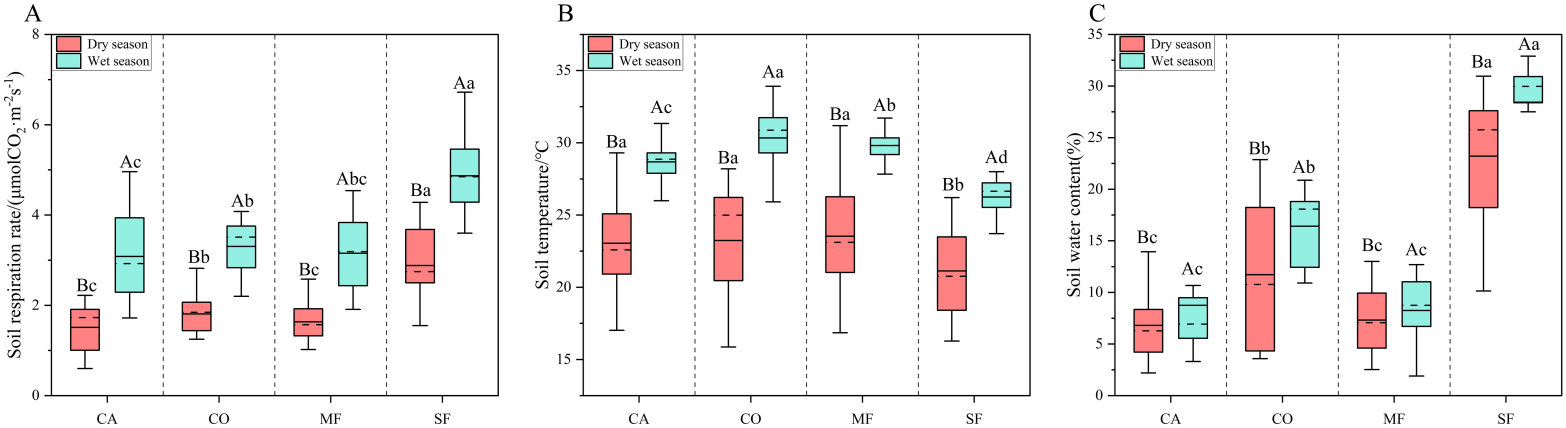
Monthly average soil respiration rate, soil temperature, and soil moisture during the dry and wet seasons across different forest stands. **(A)** Soil respiration rate; **(B)** soil temperature; **(C)** soil moisture. Note: Different uppercase letters denote significant differences between wet and dry seasons within the same forest type, and different lowercase letters indicate significant differences among forest types within the same season (*P* < 0.05).

The monthly average soil respiration rates of the different stands in both the dry and wet seasons followed the order SF > CO > MF > CA. In the dry season, the soil respiration rate in SF was 59.34%, 76.43%, and 90.50% higher than that in CO, MF, and CA, respectively, and CO was significantly higher than those in MF and CA. In the wet season, SF was 47.33%, 54.43%, and 57.86% higher than CO, MF, and CA, respectively, and CO was significantly higher than CA (**Figure 2A**). Overall, SF exhibited the highest soil respiration rate, whereas CA consistently exhibited the lowest rate in both seasons.

In the dry season, the monthly average soil temperature followed the order MF > CO > CA > SF. MF, CO, and CA did not differ significantly from each other but were all significantly higher than SF, exceeding it by 2.41°C, 2.11°C, and 1.92°C, respectively. In the wet season, the monthly average soil temperature followed CO > MF > CA > SF, with significant differences among stands (**Figure 2B**). Overall, soil temperature differences were more pronounced in the wet season, and SF displayed the lowest soil temperature in both seasons.

In the dry season, the monthly average soil moisture followed SF > CO > MF > CA. SF was 11.49%, 15.89%, and 16.41% higher than CO, MF, and CA, respectively, and CO was significantly higher than that of MF and CA. In the wet season, the monthly average soil moisture followed the order SF > CO > CA > MF. SF was 11.96%, 19.62%, and 20.13% higher than those of CO, CA, and MF, respectively. CO exhibited significantly higher moisture than CA and MF (**Figure 2C**). Overall, SF showed the highest soil moisture, whereas MF and CA exhibited lower soil moisture in both seasons.

### 3.2 Seasonal variations in soil respiration rate, soil temperature, and soil moisture

**Figure 3:**
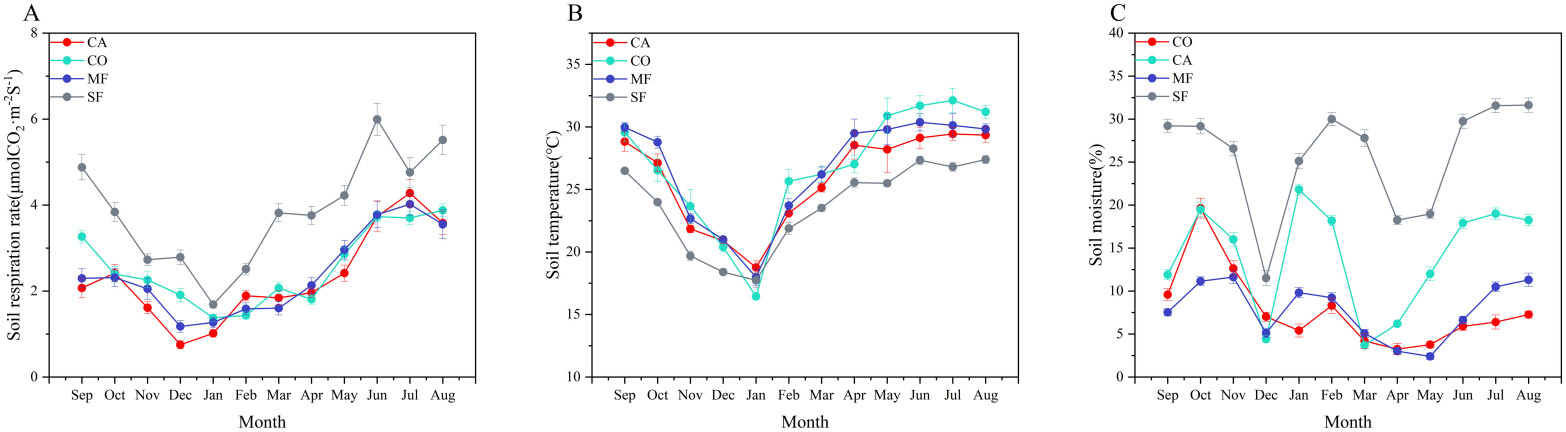
Monthly average soil respiration rate, soil temperature, and soil moisture across different forest stands from September 2023 to August 2024. **(A)** Soil respiration rate; **(B)** soil temperature; **(C)** soil moisture.

From September 2023 to August 2024, soil respiration rate, soil temperature, and soil moisture were consistently higher during the wet season (September–October 2023 and May–August 2024) and lower during the dry season (November 2023–April 2024), with significant differences observed across stands (**Figures 2**, **3**). All stands showed similar seasonal patterns in soil respiration rate, with peaks from June to August and troughs from December to January. The average soil respiration rates of CA, CO, MF, and SF in the wet season were 2.04, 1.83, 1.93, and 1.69 times higher than those in the dry season, respectively. Soil temperature also exhibited consistent seasonal variation among stands, with peaks occurring from June to August and the lowest values in January. Compared with the dry season, soil temperature in the wet season increased by 5.63°C, 7.10°C, 6.28°C, and 5.12°C for CA, CO, MF, and SF, respectively. Soil moisture exhibited a W-shaped seasonal pattern across all stands. Soil moisture levels in the wet season increased by 1.96%, 4.71%, 0.93%, and 5.18% for CA, CO, MF, and SF, respectively, compared with those in the dry season.

Repeated-measures ANOVA (**Table 3**) revealed that month, stand, and their interaction had significant effects on soil respiration rate, soil temperature, and soil moisture.

**Table 3:** Results of repeated measures ANOVA showing the effects of stand type and month on soil respiration rate, soil temperature, and soil moisture.

Project	*df*	Soil respiration rate		Soil temperature		Soil moisture	
		*F*	*P*	*F*	*P*	*F*	*P*
month	11	1752.851	<0.01	2418.842	<0.01	2736.223	<0.01
Stand	3	2191.114	<0.01	620.279	<0.01	28403.114	<0.01
month×stand type	33	67.541	<0.01	34.854	<0.01	522.098	<0.01

The annual CO_2_ emission from soil followed the order SF > CO > MF > CA. SF exceeded CO, MF, and CA by 58.82%, 64.92%, and 68.28%, respectively. There were no significant differences between MF and CA or between MF and CO, whereas CO was significantly higher than CA (**Table 4**). Overall, annual CO_2_ emission varied considerably among stands, with SF showing the highest value and CA the lowest.

**Table 4:** Regression models for soil temperature, soil moisture, and soil respiration rate, along with annual respiration flux and *Q*_10_.

Stand	*Rs=ae^bT^*			*Rs=ae^bT^W^c^*				*Rs=m+nW*			*Q_10_*	Annual flux Annual carbon fluxes/(gC·m^−2^ a^−1^)
	*a*	*b*	*R^2^*	*a*	*b*	*c*	*R^2^*	*m*	*n*	*R^2^*		
CA	0.174	0.098	0.572**	0.132	0.101	0.094	0.584**	2.35	-0.007	0.004	2.66 ± 0.11a	882.42 ± 58.78c
CO	0.357	0.071	0.698**	0.307	0.066	0.118	0.723**	2.066	0.035	0.054**	2.03 ± 0.10c	952.90 ± 49.51b
MF	0.214	0.088	0.588**	0.128	0.092	0.199	0.659**	2.043	0.045	0.018*	2.41 ± 0.11b	900.33 ± 45.56bc
SF	0.331	0.102	0.848**	0.29	0.01	0.052	0.849**	1.828	0.079	0.144**	2.77 ± 0.13a	1484.84 ± 86.11a

**P < 0.01, *P < 0.05. Different lowercase letters indicate significant differences among forest stands (*P* < 0.05).

### 3.3 Relationship between soil temperature, soil moisture, and soil respiration rate

**Figure 4:**
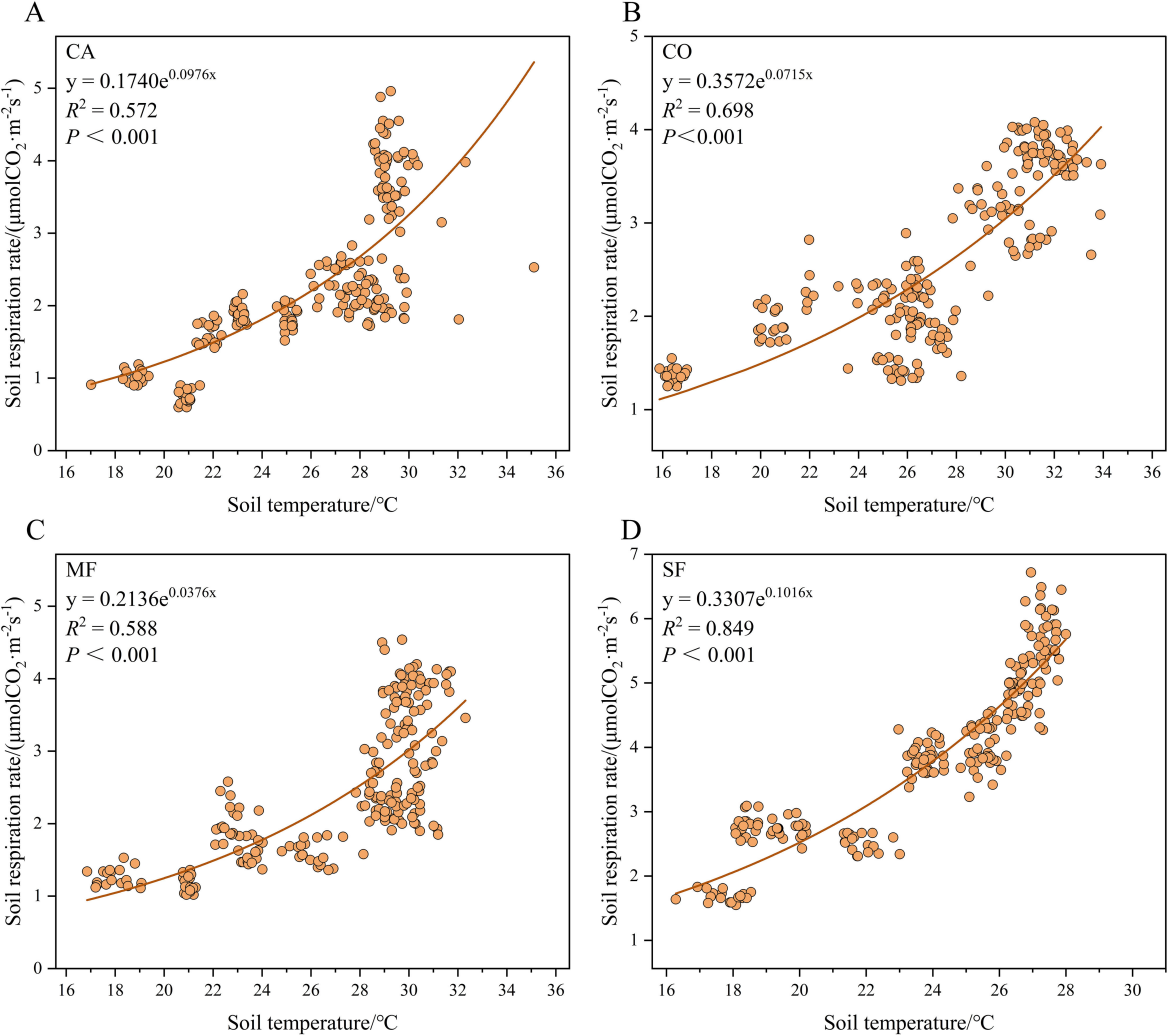
Relationship between soil respiration rate and soil temperature across the four forest types: **(A)***Casuarina equisetifolia* forest (CA), **(B)***Cocos nucifera* forest (Co), **(C)** mixed forest (MF), and **(D)** secondary forest (SF).

**Figure 5:**
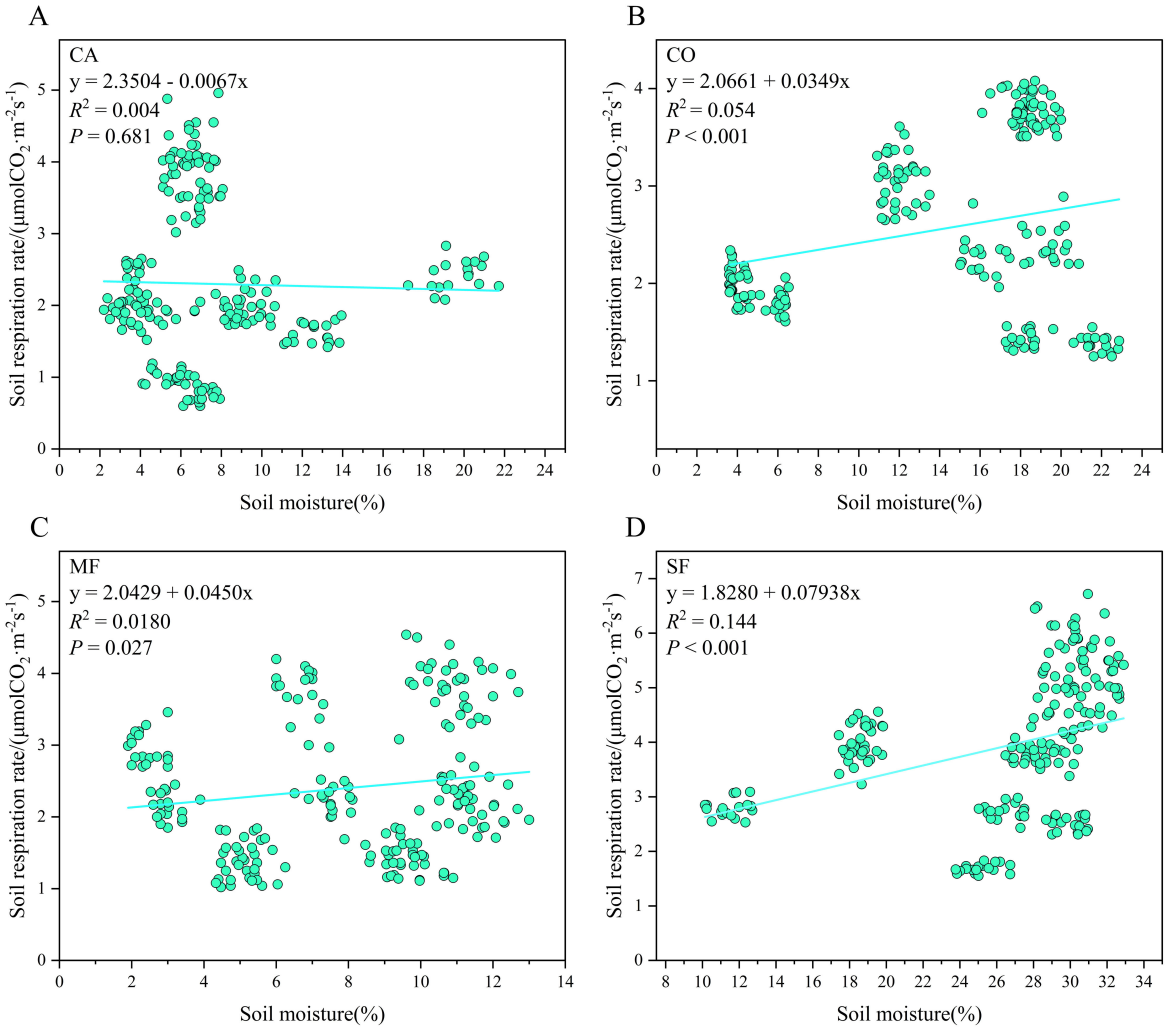
Relationship between soil respiration rate and soil moisture across the four forest types: **(A)***Casuarina equisetifolia* forest (CA), **(B)***Cocos nucifera* forest (Co), **(C)** mixed forest (MF), and **(D)** secondary forest (SF).

According to **Figure 4**, soil respiration rate and soil temperature exhibited a highly significant exponential correlation across all stands. As shown in **Figure 5**, no significant linear correlation was detected between soil respiration rate and soil moisture in CA. However, significant or highly significant linear correlations were observed for CO, MF, and SF.

Single-factor and dual-factor regression models were used to examine the correlations between soil respiration rate, soil temperature, and soil moisture (**Table 4**). In the single-factor model, soil temperature explained 57.2%, 69.8%, 58.8%, and 84.8% of the variation in soil respiration rates for CA, CO, MF, and SF, respectively, showing highly significant positive correlations (*P* < 0.01). In contrast, soil moisture explained only 5.4%, 1.8%, and 14.4% of the variation in soil respiration rates for CO, MF, and SF, respectively, and the correlation between soil respiration rate and soil moisture was not significant for CA. When the two-factor regression model was applied, soil temperature and soil moisture together explained 58.4%, 72.3%, 65.9%, and 84.9% of the variation in soil respiration rates for CA, CO, MF, and SF, respectively. These values were similar to those obtained for soil temperature alone, suggesting that seasonal variations in soil respiration rates were primarily driven by soil temperature.

The soil *Q*_10_ values among the stands followed the order SF > CA > MF > CO. No significant difference was found between SF and CA, but both were significantly higher than those of MF and CO. MF also exhibited a significantly higher *Q*_10_ than CO (**Table 4**). These results indicate that SF and CA have similar sensitivities to temperature changes in soil respiration and that both are more temperature-sensitive than MF and CO.

### 3.4 Characteristics of environmental factors in different stands

Environmental factors for the different stands during the dry and wet seasons are listed in **Table 5**. All variables showed significant seasonal variation, and the magnitude and direction of these changes differed among stands.

**Table 5:** Environmental factors of different tropical coastal shelter forests during dry and wet seasons.

Indicator	Season	Stand			
		CA	CO	MF	SF
pH	Dry season	5.65 ± 0.16 Aa	5.32 ± 0.05 Ab	4.92 ± 0.04 Ac	5.27 ± 0.11 Ab
	Wet season	4.19 ± 0.03 Bd	5.16 ± 0.04 Bb	4.56 ± 0.04 Bc	5.43 ± 0.03 Aa
SOM (g/kg)	Dry season	6.60 ± 0.65 Ac	11.11 ± 0.70 Ab	9.82 ± 0.50 Ab	39.76 ± 1.44 Aa
	Wet season	7.23 ± 0.22 Ac	9.10 ± 0.61 Bb	9.94 ± 0.40 Ab	34.20 ± 0.54 Ba
SOC (g/kg)	Dry season	3.83 ± 0.38 Ac	6.44 ± 0.40 Ab	5.70 ± 0.29 Ab	23.06 ± 0.84 Aa
	Wet season	4.19 ± 0.13 Ad	5.28 ± 0.08 Bc	5.77 ± 0.07 Ab	19.84 ± 0.31 Ba
TN (g/kg)	Dry season	0.37 ± 0.04 Ad	0.68 ± 0.07 Ab	0.47 ± 0.04 Ac	2.41 ± 0.08 Aa
	Wet season	0.27 ± 0.03 Bd	0.49 ± 0.04 Bb	0.42 ± 0.02 Bc	1.97 ± 0.07 Ba
TP (g/kg)	Dry season	0.08 ± 0.01 Ad	0.51 ± 0.06 Ab	0.25 ± 0.03 Ac	0.91 ± 0.12 Aa
	Wet season	0.10 ± 0.02 Ad	0.59 ± 0.07 Ab	0.25 ± 0.04 Ac	0.86 ± 0.16 Aa
AN (mg/kg)	Dry season	3.98 ± 0.22 Bc	9.33 ± 0.39 Ab	4.04 ± 0.10 Bc	33.52 ± 0.59 Aa
	Wet season	6.10 ± 0.26 Ac	4.20 ± 0.06 Bd	6.55 ± 0.14 Ab	10.98 ± 0.22 Ba
AP (m g/kg)	Dry season	5.26 ± 0.04 Ad	67.86 ± 2.84 Aa	21.49 ± 1.04 Bb	9.80 ± 0.95 Ac
	Wet season	1.36 ± 0.04 Bd	67.97 ± 2.26 Aa	26.62 ± 0.71 Ab	4.05 ± 0.08 Bc
PRCP (mm)	Dry season	39.65	28.39	35.53	28.83
	Wet season	244.65	283.26	255.08	254.56
TEMP (°C)	Dry season	22.85	22.90	22.77	22.28
	Wet season	27.65	27.28	27.27	26.87
RH (%)	Dry season	80.48	79.78	80.73	82.47
	Wet season	82.95	81.50	83.43	85.32

Different uppercase letters denote significant differences between wet and dry seasons within the same forest type, whereas different lowercase letters indicate significant differences between various forest types within the same season (*P* < 0.05). RS, soil respiration rate; pH, soil pH; SOM, soil organic matter; SOC, soil organic carbon; TN, total nitrogen; TP, total phosphorus; AN, available nitrogen; AP, available phosphorus; PRCP, monthly average precipitation; TEMP, monthly average air temperature; RH, monthly average relative humidity.

In the dry season, soil pH ranged from 4.92 to 5.65, with CA showing significantly higher pH than the other stands. CO and SF did not differ significantly from each other, but both were significantly higher than MF. In the wet season, soil pH ranged from 4.19 to 5.43 and followed the order SF > CO > MF > CA, with all stands significantly differing. From the dry to wet season, soil pH decreased significantly in CA, CO, and MF, whereas SF showed a slight but nonsignificant increase.

SOM and SOC contents exhibited similar seasonal patterns across stands. In the dry season, SOM content ranged from 6.60 to 39.76 g/kg, and SOC ranged from 3.83 to 23.06 g/kg. SF had the highest content, and CA had the lowest, with CO and MF not differing significantly. In the wet season, SOM ranged from 7.23 to 34.20 g/kg and SOC ranged from 4.19 to 19.84 g/kg, with SF having the highest and CA the lowest values. Significant differences were observed among stands. From the dry to wet season, SOM and SOC increased slightly but non-significantly in CA and MF, whereas both decreased significantly in SF and CO. TN content followed the order SF > CO > MF > CA in both seasons. It ranged from 0.37 to 2.41 g/kg in the dry season and from 0.27 to 1.97 g/kg in the wet season, showing significant differences among stands. TN decreased significantly from the dry to wet season across all stands. TP content also followed the order SF > CO > MF > CA during both seasons, ranging from 0.08 to 0.91 g/kg in the dry season and from 0.10 to 0.86 g/kg in the wet season. Although TP differed significantly among stands, no significant seasonal change was detected.

For available nutrients, AN ranged from 3.98 to 33.52 mg/kg in the dry season. CA and MF did not differ significantly, but both were significantly lower than CO. In the wet season, AN followed SF > MF > CA > CO and ranged from 4.20 to 10.98 mg/kg, with significant differences among stands. From the dry to wet season, AN decreased significantly in SF and CO but increased significantly in CA and MF. AP content followed CO > MF > SF > CA in both seasons. It ranged from 5.26 to 67.86 mg/kg in the dry season and from 1.36 to 67.97 mg/kg in the wet season, showing significant differences between stands. From the dry to wet season, AP content decreased significantly in CA and SF, increased significantly in MF, and showed no significant change in CO.

Overall, soil physicochemical properties exhibited distinct seasonal responses across stands. SF generally maintained higher nutrient levels, whereas CA tended to show lower values. Seasonal changes also significantly influenced soil nutrient content.

In terms of climatic factors, monthly average precipitation increased from 28.39–39.65 mm in the dry season to 244.65–283.26 mm in the wet season, an increase of 205.00–254.87 mm. Monthly average air temperature increased from 22.28–22.90°C in the dry season to 26.87–27.65°C in the wet season, an increase of 4.38–4.80°C. Monthly average relative humidity increased from 79.78–82.47% in the dry season to 81.50–85.32% in the wet season, rising by 1.72–2.85%. These results indicate marked climatic differences between the dry and wet seasons in the study region.

### 3.5 Response of soil respiration rate to environmental factors during dry and wet seasons

**Figure 6:**
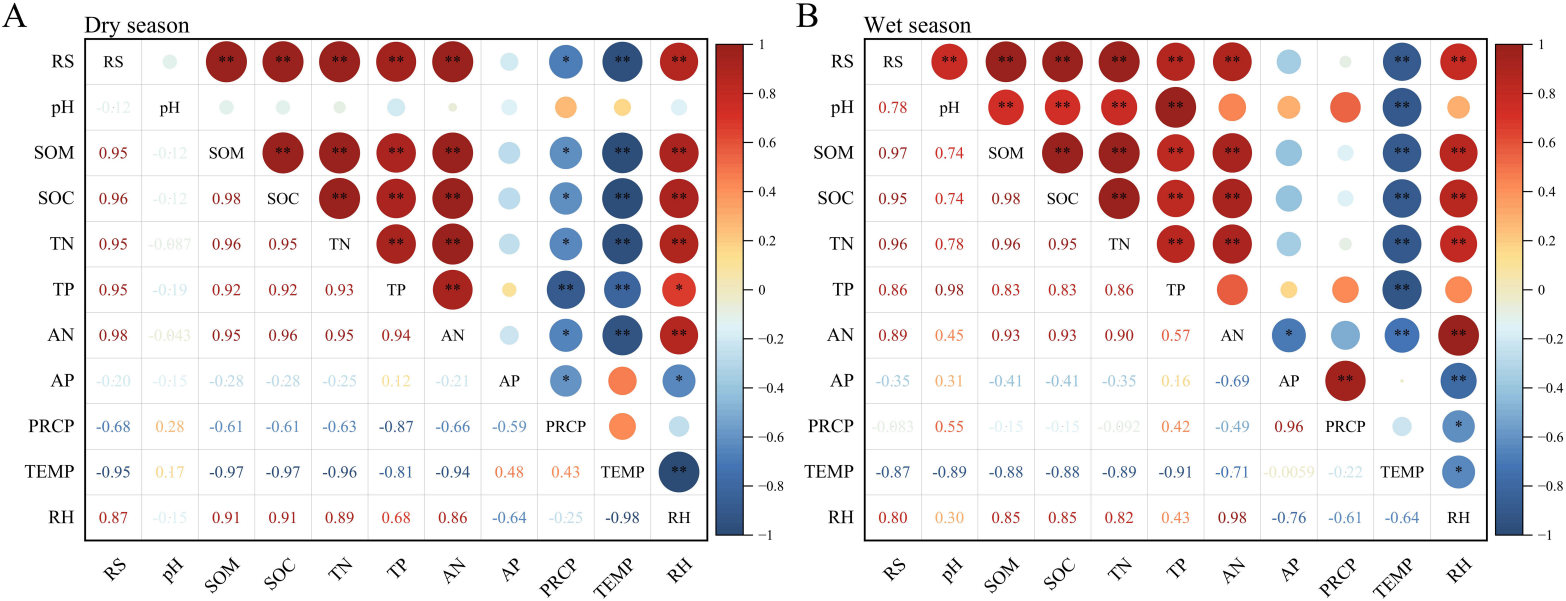
Correlation analysis between soil respiration rate and environmental factors during **(A)** dry and **(B)** wet seasons. **P < 0.01, *P < 0.05. RS, soil respiration rate; pH, soil pH; SOM, soil organic matter; SOC, soil organic carbon; TN, total nitrogen; TP, total phosphorus; AN, available nitrogen; AP, available phosphorus; PRCP, monthly average precipitation; TEMP, monthly average air temperature; RH, monthly average relative humidity.

Pearson correlation analysis was conducted between soil respiration rates and environmental factors in the dry and wet seasons (**Figure 6**). In the dry season, soil respiration rate showed extremely significant positive correlations with SOM, SOC, TN, TP, AN, and RH and significant negative correlations with PRCP and TEMP. In the wet season, the correlation between pH and soil respiration rate shifted from non-significant to highly significant positive, whereas the previously highly significant negative correlation with PRCP became non-significant. Overall, soil respiration rate was primarily influenced by nutrient availability and precipitation. In the wet season, increased precipitation strengthened the regulatory effect of soil pH on soil respiration while reducing the suppressive influence of PRCP observed in the dry season.

**Figure 7:**
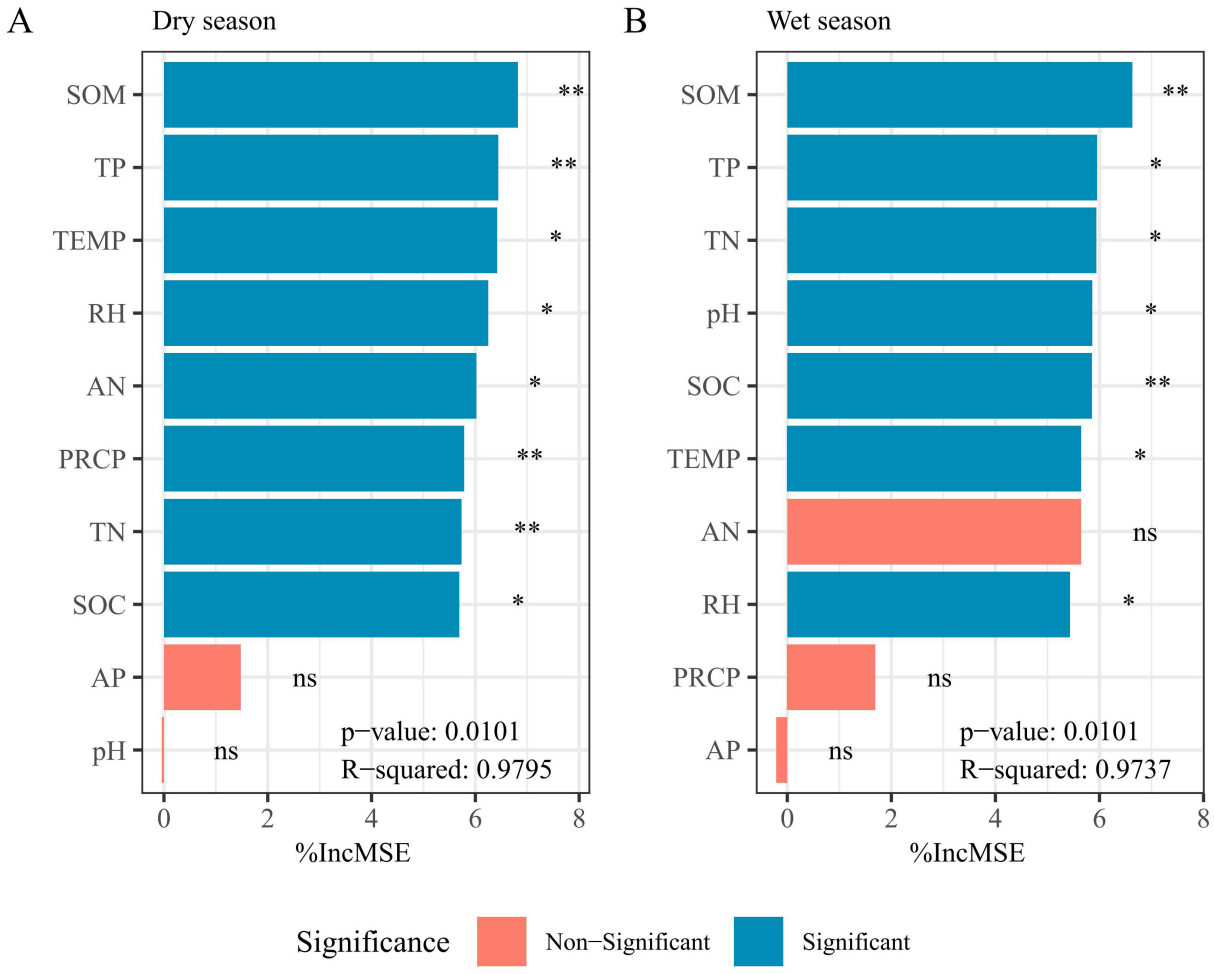
Contribution of environmental factors to soil respiration rate during **(A)** dry and **(B)** wet seasons. **P < 0.01, *P < 0.05. RS, soil respiration rate; pH, soil pH; SOM, soil organic matter; SOC, soil organic carbon; TN, total nitrogen; TP, total phosphorus; AN, available nitrogen; AP, available phosphorus; PRCP, monthly average precipitation; TEMP, monthly average air temperature; RH, monthly average relative humidity.

Based on the random forest regression model, the importance of environmental factors influencing soil respiration rates was further evaluated for both seasons (**Figure 7**). SOM was the most important factor in both the dry and wet seasons, indicating its dominant role in regulating soil respiration in tropical coastal shelter forests. TP ranked second in importance in both seasons, exerting an extremely significant influence in the dry season and a significant influence in the wet season, suggesting that TP is another key and relatively stable driver of soil respiration. The importance of PRCP was highly significant in the dry season (ranked 6th) but became insignificant in the wet season (ranked 9th). This indicates that precipitation primarily stimulated soil respiration in the dry season, whereas additional rainfall had a minimal influence in the wet season. In contrast, pH ranked low in the dry season (10th) but became a significant factor in the wet season (4th), demonstrating a stronger regulatory effect on soil respiration during the wet season. AP ranked low in both seasons, suggesting that the directly available form of phosphorus has a relatively minor direct influence on soil respiration rates.

## 4 Discussion

### 4.1 Seasonal variations in soil respiration in tropical coastal shelter forests

This study systematically revealed the typical seasonal dynamics and stand-level differences in soil respiration in tropical coastal shelter forests. Soil respiration rates were significantly higher during the wet season (May–October) than during the dry season (November–April). Peak values occurred during periods of high temperature and humidity from June to August, whereas the lowest values were recorded between December and January. This seasonal pattern is consistent with observations from tropical forests in monsoon climates (Cusack et al., 2023; Jiang et al., 2017). The mechanisms underlying this pattern reflect distinct ecological processes across seasons. During the dry season, the accumulation of forest litter and retention of SOM provide substrates that support baseline microbial metabolic activity. With the onset of the wet season, rising temperatures and humidity enhance microbial activity and accelerate organic matter decomposition, leading to higher soil respiration rates (McFarlane et al., 2024). During peak vegetation growth, vigorous root activity and associated organic carbon inputs further stimulate soil respiration (Deng et al., 2018).

In this study, annual soil respiration fluxes ranged from 882.42 to 1484.84 g C·m^−2^·a^−1^, which falls within the range reported for Chinese forests (Chen et al., 2008). These values are comparable to those recorded in subtropical coastal sandy forests in China (858–1644 g C·m^−2^·a^−1^) (Gao et al., 2018) but are lower than those reported for tropical montane rainforests in Jianfengling, Hainan Island (1567 g C·m^−2^·a^−1^) (Jiang et al., 2017). This difference is likely attributed to the nutrient-poor sandy soils characteristic of coastal shelter forests, which constrain microbial metabolism and limit annual soil respiration fluxes (Nong et al., 2025; Gao et al., 2024).

Approximately 50% of the total soil respiration originates from SOC mineralization (Hanson et al., 2000; Subke et al., 2006). In this study, SOC content followed the order SF (23.06 g/kg) > CO (6.44 g/kg) > MF (5.69 g/kg) > CA (3.83 g/kg), which closely corresponded with the patterns observed in soil respiration rates and annual CO_2_ emissions. SF exhibited significantly higher monthly respiration rates and annual respiration than the other stands. Compared with plantation stands, secondary forests typically have higher biomass, greater litter input, and more complex community structures. These characteristics enhance substrate supply and microbial activity, resulting in higher soil respiration rates and annual CO_2_ release (Chen et al., 2017; Gao et al., 2024; Chen et al., 2014; Cui et al., 2016). In contrast, CA exhibited the lowest soil respiration rate and annual CO_2_ emission. This can be attributed to the slower decomposition rate of *Casuarina equisetifolia* litter and the re-translocation of nutrients to new organs before leaf senescence (Ge et al., 2019; Xu et al., 2022). These processes limit SOC accumulation, consequently suppressing soil respiration and carbon emission.

An additional noteworthy pattern observed in this study is that SF exhibited the lowest soil temperature but the highest soil respiration rates and annual fluxes. Although this appears to contradict the typical positive correlation between temperature and soil respiration, several interacting mechanisms explain this phenomenon. First, the high SOM content in SF provides abundant active substrates for microbial metabolism, partially offsetting temperature constraints and sustaining high heterotrophic respiration (Chen et al., 2014; Cui et al., 2016). Second, thick litter layers in SF offer strong insulation and moisture retention. This decouples the microclimate that supports microbial activity beneath the litter from the measured surface soil temperature, allowing microbial processes to remain active even when surface temperatures are low (Zhuang et al., 2023). Third, the complex stand structure of SF is associated with extensive root systems, increasing the contribution of autotrophic respiration to total soil respiration (Bréchet et al., 2011). Finally, the relatively complex stand structure of SF (e.g., closed canopy and dense understory vegetation) helps create a stable and humid microclimate within the forest. Although this reduces direct solar radiation and lowers soil temperatures, it simultaneously provides conditions that favor biological activity (Gao et al., 2024). In summary, the “low-temperature, high-respiration” phenomenon observed in SF is the result of multiple synergistic mechanisms, including high substrate availability, physical buffering by the litter layer, substantial root respiration, and microclimate regulation by stand structure. These findings underscore the importance of jointly considering biotic and abiotic interactions when interpreting soil carbon fluxes.

### 4.2 Relationship between soil respiration, soil temperature, and soil moisture in tropical coastal shelter forests

Soil temperature and soil moisture are widely recognized as the most important factors influencing soil respiration (Luo and Zhou, 2006). Soil temperature regulates soil respiration by affecting microbial metabolism, root activity, and the rate of organic matter decomposition. In contrast, soil moisture influences the microbial environment and diffusion of substrates and oxygen, which determine respiration processes (Guan et al., 2024; Domeignoz-Horta et al., 2023; de Dato et al., 2017; Zhang et al., 2023). In this study, soil respiration rate was primarily driven by soil temperature, and the two variables showed a highly significant exponential positive correlation. This finding is consistent with previous studies (Gao et al., 2018; Sheng et al., 2010; Song et al., 2013). The observed pattern reflects seasonal changes in plant photosynthesis and root metabolic activity associated with fluctuations in soil temperature, resulting in higher soil respiration rates during the wet season and lower values during the dry season. Soil temperature explained 57.2%, 69.8%, 58.8%, and 84.8% of the variation in soil respiration rates for CA, CO, MF, and SF, respectively. Differences in the explanatory power among stands may be related to variations in substrate availability, root activity, and microbial activity (Chen et al., 2010).

*Q*_10_ is a key indicator of the temperature sensitivity of soil respiration (Raich and Schlesinger, 1992). In this study, *Q*_10_ values ranged from 2.03 to 2.77, with an average of 2.47. This value is close to the global average of 2.4 (Raich and Schlesinger, 1992) and within the range reported for Chinese forest soils (1.33–5.33) (Chen et al., 2008). Compared with other forest ecosystems in China, the *Q*_10_ value in tropical coastal shelter forests was slightly higher than that of subtropical coastal forests (Gao et al., 2018) but lower than that of tropical rainforests on Hainan Island (2.7) (Jiang et al., 2017). These results suggest that soil respiration in tropical coastal shelter forests is more sensitive to temperature changes than in subtropical coastal forests. However, when contrasted with well-preserved tropical rainforests, soil respiration in tropical coastal shelter forests shows relatively low temperature sensitivity. This difference is likely driven by fundamental variations in substrate availability, microbial community dynamics, and environmental controls across these ecosystems (Zhuang et al., 2023; Yang et al., 2023). Compared with subtropical coastal forests, the warmer and more humid climate typical of tropical regions promotes the accumulation and rapid turnover of labile organic carbon, potentially amplifying the response of soil respiration to warming (Li et al., 2020). In contrast to structurally complex and climatically stable primary tropical rainforests, coastal shelterbelt soils may contain younger carbon pools with proportionally higher concentrations of readily decomposable materials (Li et al., 2023). Concurrently, their microbial communities may exhibit partial thermal adaptation owing to prolonged exposure to more variable environmental conditions, resulting in temperature sensitivity values that do not reach those observed in primary rainforest ecosystems (Zhang et al., 2023). In this study, SF exhibited the highest *Q*_10_, indicating a stronger sensitivity of soil respiration to temperature fluctuations. Ecosystems with higher SOC content often show higher *Q*_10_ values because abundant SOC provides ample substrates for microbial respiration (Zhang et al., 2020; Talmon et al., 2011). Thus, the elevated SOC content in SF may be a key factor contributing to the highest *Q*_10_. Projected warming may alter carbon source–sink dynamics in shelterbelt forests by accelerating microbial decomposition and modifying substrate availability. Such effects are likely to be especially pronounced in SOM-rich ecosystems, such as SF, where warming may intensify carbon turnover (Wang et al., 2015).

Previous studies have shown that soil moisture can explain 20–50% of the variation in soil respiration rates. However, these studies primarily focused on arid regions or years with low rainfall (Luo and Zhou, 2006; Yang et al., 2005). In this study, significant linear correlations between soil respiration rate and soil moisture were observed in CO, MF, and SF, but soil moisture explained only 5.4%, 1.8%, and 14.4% of the variation, respectively. This weak explanatory power may reflect the dominant regulatory role of soil temperature, which can diminish the influence of soil moisture at the seasonal scale (Liu et al., 2019; Luan et al., 2012). Although soil temperature was the primary driver of soil respiration, bivariate regression models that incorporated both soil temperature and soil moisture improved the explanatory power. This indicates that these two factors exert interactive or complementary effects on the regulation of soil respiration in tropical coastal shelter forests (Gao et al., 2018; Hursh et al., 2017; He et al., 2024). These results further highlight the importance of water–heat interactions in shaping soil respiration dynamics.

### 4.3 Response of soil respiration rate in tropical coastal shelter forests to environmental factors

In tropical coastal shelter forests, soil respiration rates were primarily influenced by SOM and TP in both the dry and wet seasons, with SOM serving as the dominant regulatory factor. This finding is consistent with those of previous studies (Zhang et al., 2020; Li et al., 2003; Han et al., 2024). SOM acts as the main carbon source for soil respiration and supports microbial metabolism by providing substrates, regulating microenvironmental conditions, and mediating biological interactions (Moinet et al., 2016). P is also an important factor controlling soil respiration, particularly in P-deficient tropical and subtropical ecosystems (Li et al., 2021; Zhao et al., 2014). In the study area, there is pronounced P limitation, and plant growth strongly depends on P availability. Consequently, even minor fluctuations in soil P content can significantly affect soil respiration rates (Li et al., 2021; Nong et al., 2025). However, AP was not significantly correlated with soil respiration in either the dry or wet seasons, and variable importance analyses consistently ranked AP as low and non-significant. This may be because most soil P exists in insoluble forms, limiting the direct influence of AP on microbial activity and carbon cycling processes.

Seasonal changes also strongly affected the roles of PRCP and pH in the regulation of soil respiration. In the dry season, PRCP suppressed respiration by intensifying soil water stress. In contrast, during the wet season, as soil moisture approached saturation, the inhibitory effect of PRCP diminished. Meanwhile, persistently moist conditions activated pH-sensitive microbial processes. As a result, soil pH, which had no significant effect during the dry season, was strongly and positively associated with soil respiration in the wet season. Thus, seasonal shifts in PRCP and pH were the key drivers of the observed changes in soil respiration patterns. Once soil moisture reaches saturation in the wet season, precipitation ceases to be a limiting factor, and the dominant control shifts to other variables, such as pH (Metz et al., 2025). Under high moisture conditions, anaerobic microenvironments may form, increasing microbial sensitivity to acidity and strengthening the regulatory effect of pH on soil respiration (Liu et al., 2023).

Previous studies have shown that the influence of precipitation on soil respiration is generally stronger in the dry season than in the wet season (Cregger et al., 2012; Feng et al., 2024; Li et al., 2024). The present study supports this conclusion. Soil pH primarily influences CO_2_ emissions by regulating microbial activity (Shi et al., 2010). During the dry season, microbial communities often remain dormant or metabolically inactive and show limited responses to pH changes. In the wet season, abundant moisture promotes microbial physiological activity and enhances their sensitivity to pH (Evans and Wallenstein, 2012). Moreover, microbial abundance and diversity are positively correlated with soil pH (Rousk et al., 2010), which helps explain the significant positive relationship between pH and soil respiration rate in the wet season.

Although this study revealed fundamental patterns of soil respiration responses to environmental factors in tropical coastal shelter forests, certain limitations remain. For example, microbial mechanisms were not directly assessed, and the static sampling strategy may not have fully captured rapid respiration responses following precipitation pulse events. Additionally, the temporal mismatch between soil physicochemical measurements and year-round respiration data may affect the interpretation of the model results. Future research should integrate *in situ* high-frequency flux monitoring with microbial metagenomic analysis to more thoroughly elucidate the dynamic mechanisms through which environmental factors regulate soil respiration in tropical coastal shelter forests.

## 5 Conclusions

Different tropical coastal shelter forest stands exhibited distinct soil respiration rates. The monthly average respiration rate and annual flux of SF were significantly higher than those of CA, CO, and MF. Although the four stands differed in magnitude, they exhibited similar seasonal patterns. Soil respiration was significantly higher in the wet season (May–October) than in the dry season (November–April), with peak values occurring from June to August and the lowest values from December to January. Soil temperature was the dominant factor regulating seasonal variation in soil respiration, explaining 57.2% to 84.8% of the variation among stands. Soil temperature and soil moisture also exhibited a synergistic effect in the two-factor regression model. Soil organic matter was the key driver controlling soil respiration across stands, whereas seasonal changes in precipitation and soil pH were additional important contributors to seasonal variation. These multifactor interactions enrich our theoretical understanding of soil carbon cycle adaptation in tropical coastal shelter forests and underscore the need to consider both biotic and abiotic influences in ecological process research. From a management perspective, integrating the seasonal dynamics of key drivers, such as soil temperature and soil organic matter, into management strategies may help better balance ecological protection and carbon sequestration functions in these unique coastal shelter forests.

## Data Availability

The original contributions presented in the study are included in the article/supplementary material. Further inquiries can be directed to the corresponding author/s.
